# Longitudinal Deformation of Pulmonary Artery: A Case Series Study

**Published:** 2013-12-01

**Authors:** Ali Hosseinsabet

**Affiliations:** 1 Cardiology Department, Tehran Heart Center, Tehran University of Medical Sciences, Tehran, IR Iran

**Keywords:** Pulmonary Artery, Doppler Echocardiography

## Abstract

**Objectives:**

In this study, pulmonary artery longitudinal deformation was evaluated and attempted to clarify pulmonary artery deformation.

**Methods:**

Eight healthy men and seven healthy women with structurally normal hearts were enrolled. In the parasternal short axis view at the aortic valve level, longitudinal axis of pulmonary artery was obtained and color coded Doppler myocardial imaging applied.

**Results:**

The systolic strain was positive in most the pulmonary segments, velocity and strain rate waves had three defined peaks, one positive in systole and two negative in diastole.

**Conclusions:**

This study revealed for first time, deformation pattern of pulmonary artery by color coded Doppler myocardial imaging and can be basis for future researches in cardiopulmonary diseases.

## 1. Background

Up to now, in vitro researches have investigated the longitudinal deformation of Pulmonary Artery (PA) (1). Increased PA stiffness may impose a higher after load on the Right Ventricle (RV) and contribute to RV dysfunction (2). In large arteries, during systole and diastole, movement direction of inner layers of arterial wall is in radial and longitudinal direction (3). The present research aimed to evaluate the longitudinal deformation of PA in vivo by tissue Doppler echocardiography.

## 2. Patients and Methods

In this study, eight healthy men and seven healthy women (blood pressure < 140 / 80, body mass index < 25 Kg / m^2^) with structurally normal hearts and estimated systolic PA pressure of less than 30 mmHg were imaged with Vingmed Vivid 7 system after obtaining verbal consents. Three consecutive cardiac cycles were recorded during expiration with 2 - dimensional color coded Doppler myocardial imaging format from the PA in the parasternal short axis view at the aortic valve level. Then, regional velocities, strain, and strain rates were estimated by Region of Interest (ROI) area of 6X4 mm. ROI was placed in proximal, mid, and distal parts of the medial and lateral walls of PA. The timing of end-systole and end-diastole were derived by PA flow velocity profile.

## 3. Results

**Figure 1.**
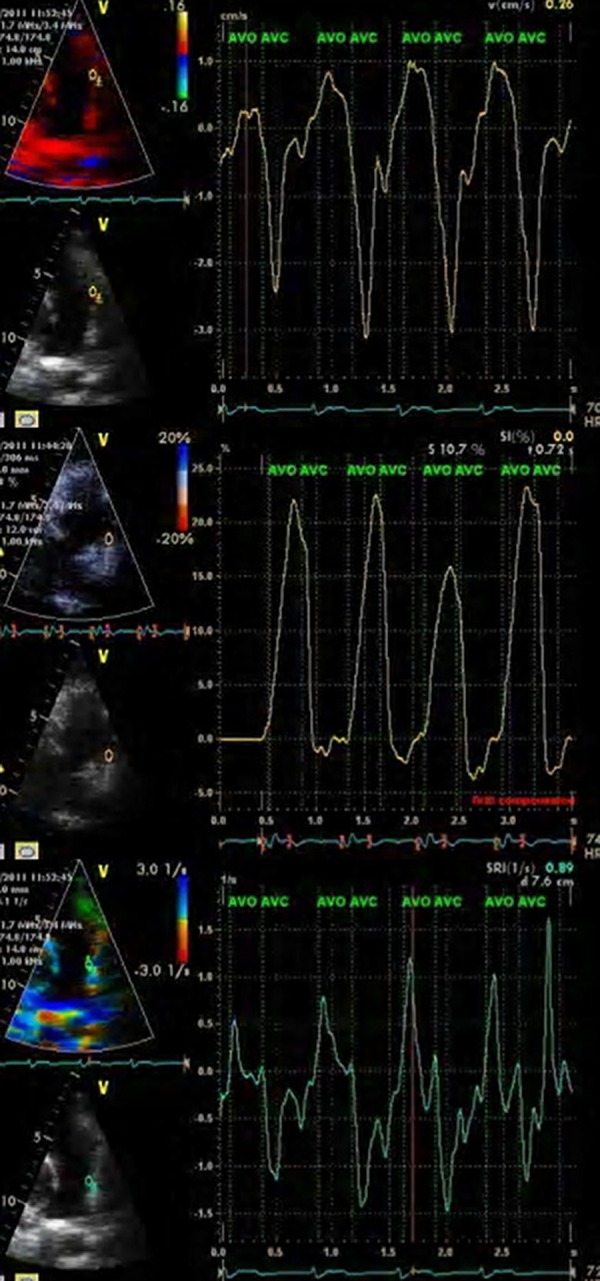
Velocity Waves (Upper), Strain Wave (Middle), and Strain Rate Waves (Lower)

The systolic strain was positive in all the pulmonary segments, except for some proximal parts of PA in the medial and lateral walls. Moreover, velocity and strain rate waves had three defined peaks, one positive in systole and two negative in diastole. However, the strain rate waves were reversed in the negative systolic strain segments (Figure 1). Nonetheless, the study results revealed no difference between the two PA walls regarding the pattern of velocity, strain, and strain rate. The results have been presented in Table 1. 

**Table 1.** Velocities, Strain, and Strain Rates of Pulmonary Artery Segments^*^[Table-fn fn6817]

Indecis Velocities (cm / s)	Sex	PL	ML	DL	PM	MM	DM
**S**	M	5.79 ± 1.73	6.97 ± 2.85	5.08 ± 2.10	6.81 ± 1.89	5.60 ± 1.37	4.98 ± 0.89
	range	(4.47_7.75)	(3.67_11.59)	(2.67_8.87)	(4.26_9.45)	(3.56_7.34)	(3.33_6.12)
	F	5.28 ± 2.07	5.69 ± 1.73	4.29 ± 1.92	5.86 ± 1.50	5.54 ± 1.27	5.11 ± 1.01
	range	(2.38_7.93)	(2.94_8.71)	(0.61_7.74)	(4.26_8.01)	(3.81_8.51)	(3.88_6.67)
**E**	M	-4.45 ± 1.75	-4.24 ± 1.58	-4.54 ± 1.40	-5.82 ± 1.37	-5.23 ± 1.49	-5.69 ± 1.47
	range	(-6.55_-3.39)	(-6.02_-2.16)	(-7.59_-1.99)	(-7.85_-4.26)	(-3.39_-8.11)	(-7.81_-3.73)
	F	-5.18 ± 3.73	-6.36 ± 2.25	-4.79 ± 1.65	-6.21 ± 1.11	-6.93 ± 2.09	-6.23 ± 1.81
	range	(-8.83_-1.07)	(-8.83_-2.29)	(-6.79_-0.86)	(-8.26_-4.77)	(-3.81_-9.14)	(-8.60_-3.69)
**A**	M	-2.81 ± 1.46	-3.12 ± 1.46	-4.53 ± 2.65	-5.01 ± 1.37	-4.52 ± 0.92	-4.25 ± 0.63
	range	(-4.10_-1.21)	(-4.74_-1.05)	(-9.95_-1.02)	(-7.61_-3.70)	(-6.06_-3.30)	(-5.42_-3.76)
	F	-2.67 ± 1.01	-5.06 ± 1.28	-4.43 ± 1.63	-5.19 ± 0.97	-5.49 ± 0.96	-4.47 ± 0.61
	range	(-4.29_-1.69)	(-8.11_-3.60)	(-7.01_-0.99)	(-6.50_-3.83)	(-6.82_-4.45)	(-5.66_-3.90)
**Strain (%)**	M	22.64 ± 7.53	16.84 ± 7.04	34.45 ± 16.64	21.91 ± 14.99	19.65 ± 11.88	22.23 ± 8.70
	range	(16.24_30.94)	(10.07_30.22)	(18.88_52.64)	(5.67_45.41)	(1.73_37.90)	(13.29_41.03)
	F	20.01 ± 7.15	18.42 ± 9.01	37.82 ± 27.08	23.20 ± 10.10	19.21 ± 10.04	28.24 ± 20.75
	range	(13.72_32.04)	(5.68_36.92)	(10.54_98.32)	(15.12_42.41)	(8.70_38.45)	(11.84_81.30)
**Strain Rates (1 / s)**							
**SRs**	M	1.32 ± 0.79	2.04 ± 0.95	2.36 ± 0.66	1.68 ± 0.97	1.37 ± 0.59	1.22 ± 0.43
	range	(0.77_2.24)	(1.28_3.85)	(1.16_3.51)	(0.88_3.41)	(0.49_2.36)	(0.63_2.20)
	F	2.33 ± 1.90	1.75 ± 0.81	2.68 ± 1.00	1.67 ± 0.57	1.49 ± 0.48	1.71 ± 0.73
	range	(0.95_5.66)	(0.68_3.26)	(1.48_5.33)	(1.10_2.61)	(1.03_2.59)	(0.63_2.86)
**SRe**	M	-2.33 ± 1.39	-1.22 ± 0.51	-1.71 ± 0.63	-1.64 ± 0.35	-1.08 ± 0.24	-1.69 ± 1.72
	range	(-3.94_-1.40)	(-1.91_-.50)	(-2.52_-0.81)	(-1.23_-2.23)	(-1.31_-0.47)	(-5.85_-0.37)
	F	-1.68 ± 0.44	-1.48 ± 0.88	-1.88 ± 1.19	-1.71 ± 0.90	-1.74 ± 1.48	-1.09 ± 0.44
	range	(-2.26_-1.09)	(-2.28_-0.21)	(-3.81_-0.37)	(-4.99_-0.82)	(-1.91_-0.43)	(-3.20_-0.57)
**SRa**	M	-1.07 ± 0.59	-0.60 ± 0.73	-1.91 ± 1.19	-1.25 ± 0.46	-1.14 ± 0.77	-1.17 ± 1.08
	range	(-1.82_-0.38)	(-2.01_-0.05)	(-3.88_-0.65)	(-2.02_-0.73)	(-2.49_-0.34)	(-3.89_-0.23)
	F	-1.31 ± 0.85	-1.00 ± 0.67	-2.03 ± 1.32	-1.28 ± 0.80	-1.30 ± 0.61	-1.36 ± 0.71
	range	(-2.07_-0.42)	(-2.13_-0.09)	(-5.62_-0.34)	(-2.75_-0.47)	(-2.28_-0.29)	(-2.55_-0.25)

^*^Results of proximal segments with positive strain included.

Abbreviations: PL, proximal segment of lateral wall; ML, mid segment of lateral wall; DL, distal segment of lateral wall; PM, proximal segment of medial wall; MM, mid segment of medial wall; DM, distal segment of medial wall; S, systolic wave; E, early diastolic wave; A, late diastolic wave; SRs, systolic strain rate; SRe, early diastolic strain rate; SRa, late diastolic strain rate; M, male; F, female

## 4. Discussion

This study showed that the pulmonary walls movements were synchronous but reverses with the heart; while the RV was contracting, the PA was longitudinally expanding and while the RV was relaxing, the PA was longitudinally contracting. Also, the waves profile was shaped depending on the pressure changes in the lumen.

When stress is under zero, the elastic lamellae are wavy longitudinally and circumferentially in the aortic wall. During physiologic stress, on the other hand, elastic lamellae are straight, while collagen fibers are uncoiling (4). This can explain the existence of deformation in the PA. Overall, the longitudinal tension and the elastic recoil of the arterial wall, an active component from the wall itself, and the shear force from the blood flow can be effective in the longitudinal PA wall movements.

Demonstration of the significant longitudinal movement of the PA walls in vivo has the potential to improve our knowledge of the elastic properties of the PA walls, ability to detect early abnormalities in the arterial wall function, and may have implications for cardiopulmonary diseases. Yet, the results of this study should be replicated in larger studies.
